# Small Pulmonary Lesions: A Challenge for Thoracic Surgery?

**DOI:** 10.1100/tsw.2001.347

**Published:** 2001-12-15

**Authors:** Klaus Kayser, Delia Donnwald, Stefan Zink, Gian Kayser

**Affiliations:** Department of Pathology, Thoraxklinik, Amalienstr. 5, D69126 Heidelberg, Germany

**Keywords:** pulmonary hamartoma, T1 carcinoma, lymphoid hyperplasia, diagnosis, surgical treatment, prognosis

## Abstract

We analyzed the diagnosis, the potentially associated external and clinical features, and the surgical procedures of small pulmonary lesions, especially hamartomas (in relation to peripheral T1 lung carcinomas and lymphoid hyperplasia) in 103 patients who experienced enucleation or resection of pulmonary hamartomas between March 1, 1995 and December 31, 2000. The causes of surgical intervention, presurgical diagnoses, surgical procedures, location, size, and histological compartments were analyzed, as well as clinical features potentially associated with the tumors (alcohol, asbestos, smoking, and chronic lung diseases). Follow up of patients lasted for 5.5 years at maximum. For comparison, 36 patients with peripheral T1 lung carcinomas are included as well as 50 patients with lymphoid hyperplasia. The sex and age distribution of the patients with hamartomas was comparable to that of patients with lymphoid hyperplasia. About 75% of men and 55% of women were heavy smokers, with an average history of 30 and 17 pack years, respectively. In 84% of patients, the lesions were incidentally detected in chest radiographs, whereas 12% of patients underwent thoracic surgery suspicious for intrapulmonary metastases of known extrapulmonary malignancies. Enucleation was performed in 21%, and wedge resection in 77% of patients. At average, hamartomas were smaller than T1 lung carcinomas, but considerably larger in comparison to lymphoid hyperplasia. No recurrent tumors or additionally detected hamartomas were noted during the follow up, and both surgical procedures (enucleation or wedge resection) were identical in curative treatment. All patients with peripherally localized T1 tumors underwent lobectomy. The 3/5 year survival rate was calculated to 69/52%. Lymphoid hyperplasia is of clinical importance for the estimation of prognosis in patients with metastatic disease, as the number of radiologically suggestive metastatic nodules can often be significantly changed due to this entity. Pulmonary hamartomas are benign lesions that display certain clinical associations with malignant lung carcinomas in respect to external risk factors, and to lymphoid hyperplasia. Both surgical procedures (enucleation or wedge resection) can be performed, giving identical results in respect to treatment.

## INTRODUCTION

Benign tumors of the lung are rare, comprising only about 5% of all intrapulmonary tumors[1,2]. Of these, pulmonary hamartomas (also called fibro-chondro-lipoma) are the most frequently seen[1,2]. These tumors commonly possess different components, consisting of mature mesenchymal tissue, such as fat, chondroid, and fibrous tissue. The quantity of these components within the lesion varies; however, most frequently all three components are present[1,2]. As benign lesions, hamartomas possess strict boundaries and can easily be palpated during surgery[3,4]. Hamartomas are removed surgically in one of two ways: by enucleation, in which the lesion is palpated and excised without surrounding lung tissue, or by wedge resection, in which a “security string” of pulmonary tissue is excised in addition to the lesion[1,2,3]. The diagnosis can be confirmed macroscopically in the majority of lesions; however, histologic confirmation is still necessary, as these lesions can be confused with metastatic tumors such as osteo, fibro, or leiomyosarcomas[1,2,3,4].

Hamartomas seem not to possess striking clinical or surgical features at a first glance; however, they are interesting lesions in respect to their origin and their biological behavior. It is still in question whether these lesions are of neoplastic origin, or whether they are just chronic inflammatory lesions with some specific abnormalities mimicking a neoplasm[1,2]. Recently, chromosome analysis has been performed, and hamartomas often display abnormalities of the 12q15 and 6p21 regions, which has been suggested indicates a similarity to proliferation and mesenchymal tumors[5,6]. Histochemical investigations could confirm a mesenchymal neoplasm and a certain “neighborhood” of the soft-tissue compartments within these lesions[7]. However, the epithelial compartments are not considered to be related to the mesenchymal tumor growth, and it is also persuasive to postulate the origin of these tumors from some interstitial lung tissue altered by chronic interstitial inflammation[1,2,3,4]. The two theories do not necessarily exclude each other; that is, the lesion could be triggered by chronic interstitial inflammation and proceed to a slowly growing benign neoplasm. Cytogenic and histochemical investigations could not definitely solve this problem. Because hamartomas are small, peripheral lung lesions, it might be useful to include clinical aspects and different small lesions in this discussion; for example, peripherally localized small-lung carcinomas (T1N0 stage) and the frequently occurring, nonmalignant lymphoid hyperplasia. Lymphoid hyperplasia is a noncapsulated pseudo-lymph node that often mimics metastatic intrapulmonary tumor growth in computed tomography; such a misinterpretation can seriously affect the patient’s fate. Thus, the clinical differential diagnoses of hamartomas include small carcinomas and lymphoid hyperplasia, despite metastases in patients with known extrapulmonary cancer.

Our prospective study was designed primarily to contribute to chromosome analysis of deep-frozen hamartomatous tissue. It soon became clear that clinical features, patient behavior, and prognosis might make an equally important, if not superior, contribution to the increase of knowledge of this rare entity of a benign lung lesion.

## MATERIAL AND METHODS

This prospective study started March 1, 1995 and includes all patients with surgically excised pulmonary hamartomas treated at the Thoraxklinik, Heidelberg, until December 31, 2000. A total of 103 patients were included, of whom 45 underwent a preoperative transbronchial biopsy. A definitive diagnosis could not be obtained by these preoperative investigations, probably due to sampling errors (i.e., missing the lesion). For comparison, a contemporary prospective study including 36 patients with radiologically comparable lesions (staged T1 N0) resulting in histologically proven primary lung carcinomas was included in this analysis. The third cohort comprises 50 patients with peripheral lesions suspicious for malignancy or metastatic growth, and histologically confirmed as lymphoid hyperplasia. The diagnosis in all cohorts is based on

**TABLE 1**
**Synopsis of Material in Relation to Lung Carcinomas**

| | Hamartomas | | T1 Carcinomas | | Lymphoid Hyperplasia | |
|---|---|---|---|---|---|---|
| Number of cases | 103 | | 36 | | 50 | |
| Men | 61 | | 24 | | 29 | |
| Mean age | 55 ± 10 | | 64 ± 9 | | 56 ± 9 | |
| Women | 42 | | 12 | | 21 | |
| Mean age | 53 + 12 | | 63 ±11 | | 54 ± 10 | |
| Men/women | 1.5/1 | | 2.0/1 | | 1.4/1 | |
| Smokers | | Mean py | | Mean py | | Mean py |
| Men | 46/61 (75%) | 30 | 24/20 (83%) | 34 | 10/29 | 23 |
| Women | 23/42 (55%) | 17 | 12/7 (65%) | 21 | 6/12 | 14 |

surgical specimens only, and includes common HE stains, PAS, and Feulgen stains. The history of the patients was analyzed, with specific attention given to smoking and alcohol consumption. Information about surgical procedures, potential side symptoms, and the presence of other diseases was obtained from the intraoperative reports. The clinical trial was determined January 31, 2001. Survival was evaluated by repeated questionnaires sent to the house physicians. The statistical analysis was performed using a commercially available package (Number Cruncher Statistical System, NCSS, Kaysville, UT).

## RESULTS

A total of 103 patients with hamartomas were included in this study, including 61 men and 42 women (Table 1). The mean age was computed to 53 years (range 33 to 86 years) in men and to 51 years (18 to 69 years) in women (Fig. 1), which is the same range of patients with lymphoid hyperplasia. In contrast, patients with T1 lung carcinomas were older (Table 1). In the hamartoma cohort, the percentage of smokers was 75.4% in men and 54.8% in women, with a total of 29.7 pack years (py) in men and 16.8 py in women, at average (Fig. 2). These figures are in close agreement with those of common lung cancer patients in our hospital[8,9], and to those of the T1 lung cancer cohort (Table 1). The patients displaying lymphoid hyperplasia were less frequent and less heavy smokers (Table 1). Adenocarcinomas were the predominant cell type of the T1 carcinomas, which already displayed lymph node metastases in 9/36 cases (25%, Table 2). The majority of the lesions in all studies were accidentally detected by chest radiographs taken for other reasons, such as screening for metastases, preoperative chest x-rays to exclude any additional risk of surgical intervention on other organs, and so forth (Table 3). Only five patients in the hamartoma cohort and eight patients in the T1 carcinoma cohort with a high cigarette consumption (41 py and 43 py, at average) reported severe symptoms, which led to intensive clinical investigations (eight patients with peripheral lung cancer and none with lymphoid hyperplasia, Table 3). The localization of the hamartomas was found to be equally distributed in respect to the upper and lower lobes (Fig. 3) which was also seen in the patients with T1 carcinomas and lymphoid hyperplasia (not shown). Commonly, hamartomas are peripheral lesions adjacent to or at minimum distance from the pleura, and only ten hamartomas were localized in the central parts of the lung (by definition, none of the T1 carcinomas and none of the lymphoid hyperplasia, Fig. 3). Therefore, wedge resection and enucleation were the surgical strategies, and only two hamartomas required a lobe resection (Fig. 3). Hamartomas

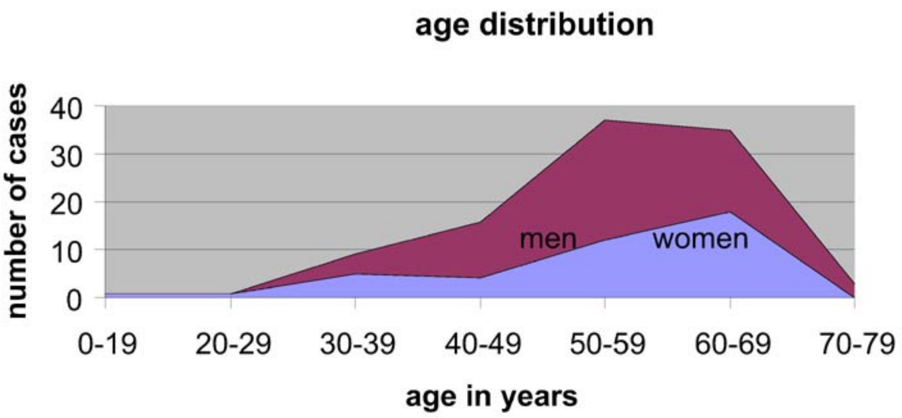

**FIGURE 1.** Age distribution of 103 patients with pulmonary hamartomas.

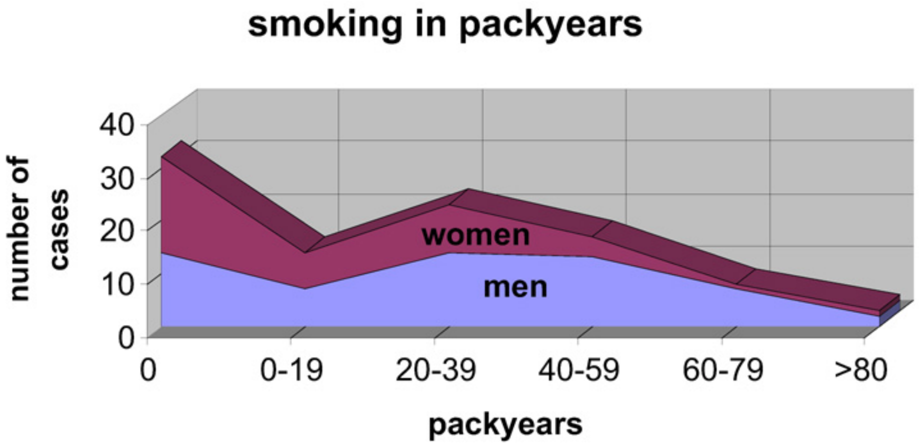

**FIGURE 2.** Amount of cigarette smoking of patients with pulmonary hamartomas (in pack years).

**TABLE 2**
**Cell Type and Postsurgical Stage of T1 Lung Carcinomas**

***Cell Type***

| | | |
|---|---|---|
| Epidermoid | 10 (28 %) | 8 men, 2 women |
| Adeno | 26 (72 %) | 16 men, 10 women |

***Cell Type and pN Stages***

| | Adeno | Epidermoid | Total | 3-Year Survival | 5-Year Survival |
|---|---|---|---|---|---|
| N-0 | 17 | 10 | 27 (75%) | 81 % | 63 % |
| N-+ | 9 | 0 | 9 (25%) | 33 % | 20 % |
| ***Total*** | | | ***36*** | ***69 %*** | ***52 %*** |

## TABLE 3
## Detection of Lesions, Histopathologic Compartments, and Tumor Size of Hamartomas

| ***Detection of Lesions*** | ***Hamartomas*** | ***T1 Carcinomas*** | ***Lymphoid Hyperplasia*** |
|---|---|---|---|
| Incidentally: | 85 (24 py) | 30 (28 py) | 11 (27 py) |
| Symptoms: | 5 (41 py) | 6 (43 py) | |
| Known malignancy: | 13 (14 py) | | 39 (12 py) |

***Size of Lesions***

| | | | |
|---|---|---|---|
| Mean diameter: | 1.5 ± 0.5 cm | 1.2 ± 0.5 cm | 0.3 ± 0.1 cm |
| Mean volume: | 2.3 ± 0.8 $cm^3$ | 8.1 ± 4.0 $cm^3$ | 0.2 ± 0.1 $cm^3$ |

***Number of Tumors***

One patient had two and one patient three contemporary hamartomas
A total of 77 lymphoid hyperplasia nodules have been excised in 50 patients (1.5 nodules/patient)

***Histological Components of Hamartomas (Absolute Numbers)***

| | |
|---|---|
| Chondroid - fat - fibrous: | 93 |
| Fat-fibrous-smooth muscle: | 7 |
| Fat-fibrous: | 3 |

3a) Localization of hamartomas upper lobes : 45 %

lower lobes : 45 %

middle lobe : 10 %

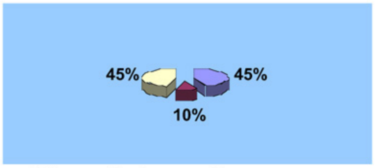

3b) surgical procedure wedge resection : 77 %

enucleation : 21 %

lobe resection 2 %

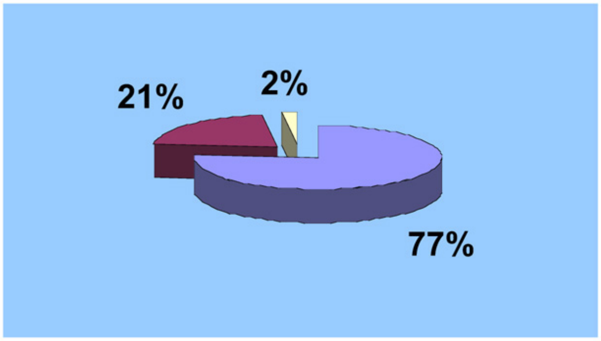

**FIGURE 3.** Localization of pulmonary hamartomas (*a*) and surgical procedures (*b*). a: hamartomas of the upper lobes, 45%; lower lobes, 45%; middle lobe, 10%. b: wedge resection, 77%; enucleation, 21%; lobe resection, 2%.

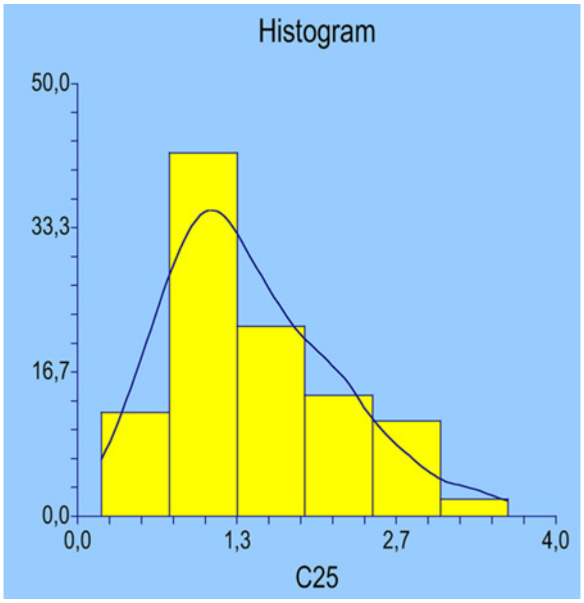

**FIGURE 4.** Distribution of size (maximum diameter, in cm) in hamartomas.

usually occur as single lung lesions, and only two patients presented with multiple hamartomas (one patient with two and one with three contemporary lesions). In contrast, lymphoid hyperplasia often occurs as multiple lesions, and about 1.5 nodules were excised in a singular patient at average. A wedge resection of T1 tumors with intraoperative tumor diagnosis and consecutive lobe resection was performed in all cases, followed by extensive lymph adenectomy. Hamartomas are generally of small size (Fig. 4), typically measuring 1.5 cm in diameter (2.3 $cm^3$ volume) at average, and are composed of chondroid, fat, and fibrous tissue in more than 90% of cases (Table 2). The patients have a favorite prognosis, and only four patients died within the follow-up period of 72 months, all of them due to lung metastases from extrapulmonary tumors.

The 36 patients with peripheral lung carcinomas show a mean age of 63.2 ± 7.2 years. The lesions were equally distributed in the upper and lower lobes, with a mean tumor volume of 8.1 ± 4.0 $cm^3$. Distribution of sex and smoking habits were comparable with the hamartoma group. The lymphoid hyperplasias were small lesions, measuring at average 0.3 cm in maximum diameter in patients with contemporary malignancies or inflammatory lesions (Table 3).

## DISCUSSION

Small, peripherally localized lesions of the lung are suspicious for malignancy in most cases, and to our experience not infrequently hinder a potential curative surgical treatment of a known lung cancer due to suggested intrapulmonary metastasis. Surprisingly, not infrequently (in our material 10 to 15%) these lesions are of benign nature, if histologically properly? diagnosed. Within this entity, hamartomas of the lung are the most frequent intrapulmonary benign lesion. Benign lung tumors contribute to about 5% to all lung tumors[1,2,3,4]. Makitaro et al.[10] reported that the majority of the benign tumors are hamartomas, with a male/female ratio of 1.25:1 in a population-based study of Northern Finland comprising 653 tumors during the years 1990 to 1992. They are still subject to diverse discussions about their nature and therapeutic strategies[3,11,12]. Being without doubt benign lesions with only a low proliferative tendency, a demand for surgical intervention seems not to exist[3,4,13,14]. On the other hand, singular reports mention the possibility of malignant transformation of large, benign, soft-tissue tumors in the lung, leading to a fatal outcome within a few months[1,2,3]. In addition, there is no live imaging technique to discriminate definitely between hamartomas and small, peripherally localized malignant lung tumors[1,2,3,4,13,14,15]. Although certain morphometric techniques, such as fractal geometry[15], can assist the diagnostic CT accuracy, the overall specificity and sensitivity of CT guided, transthoracic fine-needle biopsies to distinguish between benign and malignant lesions ranges between 85 to 90%[13]. Therefore, only surgical excision, including intraoperative histologic examination (frozen section service), can definitely exclude a malignant tumor in these lesions, especially when only an enucleation is performed[13,16]. Wedge resection and enucleation seem to be adequate techniques, especially as hamartomas are usually singular, peripherally localized lesions[1,2]. Otani et al. report that about 78% of their patients with hamartomas underwent a lobectomy or segmentectomy, and none of the afterwards excised benign tumors was resected with an extensive surgical procedure[3]. In our material, a video-assisted wedge resection was the most frequently used technical procedure, followed by enucleation, and only a few patients had to be treated by lobectomy. The majority of hamartomas are singular lesions and located in the peripheral parts of the lungs and are therefore easily reached by video-assisted surgery. Multiple lesions are often associated with Carney’s syndrome[2,17,18]. In respect to localization, no preference of the upper or lower parts of the lungs could be observed. From the morphologic point of view, they are composed of the three major soft tissue components in the majority of cases; namely, fat, chondroid, and fibrous tissue. The contribution rate of these three compartments varies; however, each commonly contributes 15 to 25% in a lesion and is easily detectable by light microscopy. Hamartomas are usually small, well-circumscribed masses, measuring 1.5 cm in maximum diameter at average. Some, however, can reach quite large sizes; the largest tumor in this series measured 4.8 cm in maximum diameter, and Fujino et al. reported a case of a hamartoma occupying the complete right lung without any severe symptoms[19].

With respect to potential risk factors of hamartomas, it might be useful to analyze living habits of the patients, such as smoking and exposure to asbestos, which are known to be associated with common lung cancer. Indeed, the majority of our patients were heavy smokers; about 75% of the men had a smoking history of 38.8 py at average, while 55% of the women had 29.7 py. These figures are similar to those reported from lung cancer patients. The sex ratio which holds in common lung cancer about 8:1 (men:women) is strikingly altered in favor to women in this series with peripherally T1 tumors (2:1) and hamartomas (1.5:1) (men:women). A chronic obstructive lung disease (COPD) was diagnosed in close relation to smoking habits in men (48% of smokers, or 38.8% of the entire cohort). However, only 8% of patients presented with an obstructive emphysema. Hamartomas rarely occur in children[20].

The prognosis of patients with hamartomas is excellent, and only four patients died within the follow-up period of 72 months. None of these four patients died due to the hamartoma, and all of them suffered from malignant tumors metastasizing into the lung. No association of the survival with the surgical technique could be observed; that is, enucleation of the hamartomas is a save and curative treatment of these lesions.

## ACKNOWLEDGMENT

The financial support of the Verein zur Förderung des biologisch-technologischen Fortschritts in der Medizin e.V. is gratefully acknowledged.
